# Optical Magnetic Induction Tomography of the Heart

**DOI:** 10.1038/srep23962

**Published:** 2016-04-04

**Authors:** Luca Marmugi, Ferruccio Renzoni

**Affiliations:** 1 Department of Physics and Astronomy, University College London, Gower Street, London WC1E 6BT, United Kingdom

## Abstract

Atrial Fibrillation (AF) affects a significant fraction of the ageing population, causing a high level of morbidity and mortality. Despite its significance, the causes of AF are still not uniquely identified. This, combined with the lack of precise diagnostic and guiding tools, makes the clinical treatment of AF sub-optimal. We identify magnetic induction tomography as the most promising technique for the investigation of the causes of fibrillation and for its clinical practice. We therefore propose a novel optical instrument based on optical atomic magnetometers, fulfilling the requirements for diagnostic mapping of the heart’s conductivity. The feasibility of the device is here discussed in view of the final application. Thanks to the potential of atomic magnetometers for miniaturisation and extreme sensitivity at room temperature, a new generation of compact and non-invasive diagnostic instrumentation, with both bedside and intra-operative operation capability, is envisioned. Possible scenarios both in clinical practice and biomedical research are then discussed. The flexibility of the system makes it promising also for application in other fields, such as neurology and oncology.

Cardiac arrhythmias are heart conditions characterised by irregular, slow, or accelerated, beating of the heart. Their causes are related to abnormal generation and conduction of the electrical pulses that control the heart’s activity^1^.

Among the most diffused sustained arrhythmias are atrial fibrillation (AF), which affects more than 10% of population over 70, and ventricular fibrillation (VF). They manifest as a turbulent cardiac activity and represent one of the most important causes of morbidity and mortality in rich countries^2^.

Nevertheless, little is known about the fundamental causes and mechanisms of fibrillation; consequently, the clinical treatment of these conditions, either via drug therapy^3^ or surgical procedure, is sub-optimal^4^. In particular, radio-frequency catheter ablation (RFCA) in AF has an estimated success rate of ~50% after 1*yr*, and multiple procedures are often required^5^.

Among the reasons of such discouraging statistics, a central role is played by the lack of: i. insight on the fundamental triggers and supporters of the fibrillation, ii. effective clinical assessment and evaluation, and iii. surgery-guiding tools. This is mainly due to the absence of tailored and effective diagnostic methods. In fact, magnetocardiography (MCG) and electrocardiography (ECG) do not provide any direct information about the causes of the irregular beat and, more importantly, the structures producing it. Recently, the first indications of localised stable sources were found in patients referred for AF, both in the frequency^6^ and in the time^7^,^8^ domains. It was then argued that fibrillation is caused by permanent modifications of the heart’s local conductivity, which produce deterministic sources known as rotors, and by the stochastic interaction with the local anatomy, which creates randomly distributed re-entry paths (wavelets). However, the topic is still widely debated^9^.

In this paper, magnetic induction tomography (MIT) is proven to be the ideal tool to investigate the causes and the mechanisms of fibrillation. In fact, it generates a non-invasive, space-resolved map of the heart’s conductivity, thus addressing directly the presence of permanent conduction anomalies and their relationship with the generation and the dynamics of AF and VF. In order to meet the requirements for such an instrument, we design an MIT imaging system based on optical atomic magnetometers (OAMs). OAMs solve the sensitivity and bandwidth issues of standard MIT systems based on pick-up coils and similar technologies, and will provide flexible, efficient and robust detection of the MIT signal, in unshielded environment and at room temperature. The proposed instrument has the potential for bedside tomography capability, with expected impact also on the screening and monitoring of patients, and in the AF surgery planning and navigation.

A novel class of non-invasive diagnostic tools, which will fulfil the need of dedicated resources for AF, VF and other conditions where electrical properties play a major role, can be thus envisioned.

## Results

### Conductivity Mapping via Magnetic Induction Tomography

Magnetic induction tomography is a recently developed technique for the investigation of the electrical and magnetic properties of an object^10^. It provides a direct mapping of conductivity *σ*, permittivity *ε* and permeability *μ*, without requiring any physical contact. It finds applications in detection of metallic objects, either for screening^11^, or for non-destructive evaluation of quality and integrity in industrial processes^12^. It was recently proposed also for the localisation of cerebral haemorrhages^13^.

**Figure 1:**
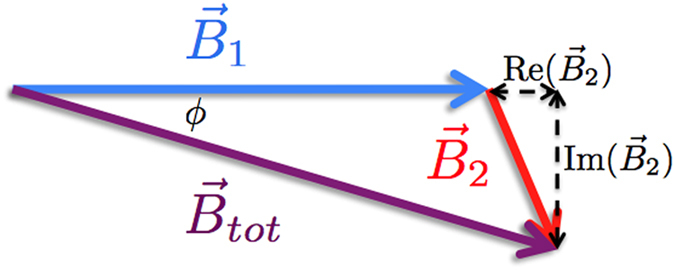
MIT principle: relationship between primary (**B**_**1**_) and secondary (**B**_**2**_) fields.

MIT exploits the response of the object of interest to an AC magnetic field (primary field, **B**_**1**_). This field excites eddy currents in the sample, which produce a secondary field (**B**_**2**_), opposing **B**_**1**_. The magnitude and phase of the total magnetic field (**B**_**tot**_, see Fig. 1) depend on the object’s electrical and magnetic properties: by performing position-resolved measurements of **B**_**tot**_, the dielectric characteristics of the sample can be effectively mapped.

If the penetration depth of the primary field is larger than the thickness of the object of interest, then the secondary field, oscillating at the frequency *ω*, can be estimated as^14^:


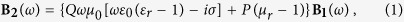


where *Q* and *P* are geometrical factors, *ε*_*r*_ is the relative permittivity, and *μ*_*r*_ is the relative permeability. Here, *ε*_*r*_, *μ*_*r*_ and the electrical conductivity depend on the object of interest; hence, the possibility of mapping its characteristics. It is worth noting that *σ* produces a purely imaginary contribution and, therefore, can be effectively isolated from other terms^14^, as described in the following.

Hence, we propose here MIT as the most promising solution for non-invasively addressing the conductivity of biological tissues and, in particular, of the heart. In fact, alternative hybrid approaches based on magneto-acoustic tomography with magnetic induction (MAT-MI) are mainly sensitive to steep changes of conductivity and rely on the coupling between large magnetic field excitation of eddy current and acoustic waves^15^. In addition, given the underlying assumption of acoustic homogeneity, they appear not suited for mechanically composite structures like the human thorax^16^.

In standard magnetic induction tomography, a driven coil produces **B**_**1**_; a set of coils then detects the magnetic field perturbed by the object of interest. However, such devices suffer from the drawbacks and limitations of the coils: indeed, the ultimate performance of MIT depends on the magnetic field sensor’s characteristics. Conventional systems therefore have limited sensitivity at low frequencies, do not provide enough bandwidth to adapt to different, non-conductive objects, and cannot be reduced below the physical size of the pick-up coil, thus limiting also the spatial resolution. In addition, they require a physical connection (i.e. wiring) with the read-out electronics and a dedicated calibration. All these facts unfortunately prevent the realisation of a working device for the MIT imaging of the heart.

### Sensitive detection with Optical Atomic Magnetometers

The limitations in sensitivity and spatial resolution of the conventional MIT devices can be overcome by using OAMs for the detection of the secondary field produced by the eddy currents.

An optical atomic magnetometer is essentially a sample of atoms, optically pumped in a known quantum state sensitive to the external magnetic field, and probed by a resonant laser. Under the influence of the external magnetic field, atoms undergo Larmor precession^17^. In an external field **B**, atomic spins precede at frequency *ω*_*L*_, directly proportional to the modulus of the magnetic field:


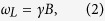


where:


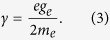


Here, *e* is the electron’s fundamental charge, *m*_*e*_ its rest mass, and *g*_*e*_ = −2(1 + *α*/(2*π*) + *o*(*α*^2^)), with *α* ≈ 1/137 the fine structure constant.

As a consequence of the atomic precession, the optical response of the atomic sample is changed. Such modifications can be detected by analysing the polarisation of a resonant probe beam: according to Eq. 2, the measurement of the magnetic field is effectively and robustly translated to a measurement of frequency.

OAMs can reach extreme sensitivity (better than 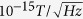), comparable to the performances of super-conductive quantum interference devices (SQUIDs)^18^. Contrary to SQUIDs, OAMs are capable of room temperature and unshielded operation, they have an extreme potential for miniaturization^19^, and they do not require any calibration, as a consequence of Eqs 2 and 3. In addition, which is particularly appealing for the MIT application discussed here, their bandwidth is not limited by intrinsic factors and their sensitivity can be up to 10^7^ times larger than that of a standard pick-up coil of the same size below 50 *MHz*^20^. Finally, OAMs do not require physical contact between the actual sensor, i.e. the atomic vapour, and the read-out electronics, and they are suitable for an array arrangement.

### OAMs-based MIT

We propose to produce the MIT primary field **B**_**1**_ with an array of coils placed above the patient’s chest, roughly ~10 *cm* above the skin, as this distance has been already demonstrated to not affect the MIT measurement^21^. Nevertheless, different positioning and arrangement are possible. In particular, the exciting coil can be integrated within a catheter and thus inserted directly in the patient’s heart during the RFCA.

**Figure 2:**
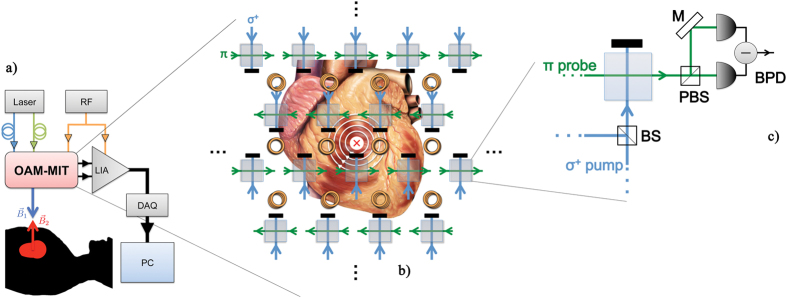
OAMs-based MIT of the heart: possible setup. (**a**) Arrangement for bedside operation. RF: radio-frequency current supply; LIA: lock-in amplifier; DAQ: data acquisition board; PC: computer. (**b**) Detailed view of the OAMs’ array and MIT coils in planar geometry. Eddy currents produced in the heart are represented as white loops. **B**_**2**_ is depicted as a vector oriented towards the heart. (**c**) Detail of the OAM sensor unit. BS: beam-splitter; PBS: polarising beam-splitter; M: mirror; BPD: balanced photodiode. Coils are not shown for simplicity. More details can be found in Methods. The heart sketch is an adaptation of an image released under Creative Commons Attribution 2.5 License 2006. Credits are to Patrick J. Lynch, medical illustrator, and C. Carl Jaffe, MD, cardiologist.

The MIT coil is driven by an AC current supply, with tuneable frequency in the range of 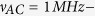 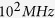. The power requirements for the current supply (RF in Fig. 2) can be easily met by commercial devices: magnetic fields of the order of *B*_1_ ≤ 10^2^ *mG*, thus much smaller than the ones required for other techniques such as MAT-MI^15^,^16^, should be sufficient for imaging. In the endoscopic arrangement, one can also take advantage of the same source used for the RFCA, by suitably decreasing its power for the imaging and then increasing it up to 50 *W* for the scarring procedure^4^.

The total magnetic field **B**_**tot**_ is detected by an array of optically pumped radio-frequency (RF) OAMs^22^. Different setups can be designed^23^,^24^,^25^ and effectively integrated in the MIT system. However, we propose here the arrangement with the least stringent requirements and the smallest footprint. The core of the measuring apparatus is thus an array of RF atomic magnetometers, operating in MIT modality. A sketch of the proposed setup is shown in Fig. 2, in the case of the simple planar geometry.

After the interaction with the preceding atomic vapour, the probe beam’s polarisation is rotated, with the polarisation oscillating at the Larmor frequency. The measurement of **B**_**tot**_ and hence of **B**_**2**_, related to the local conductivity of the heart, is effectively and robustly reduced to the measurement of the probe’s polarisation oscillation. A balanced photodiode (see Fig. 2) for each cell of array generates a correspondingly modulated electrical signal.

The output of the OAMs is selectively amplified by a dual-phase, multi-channel lock-in amplifier, referenced to the RF waveform driving the primary field. This allows rejection of the background noise and thus detection, in unscreened environments, of the contributions due to the secondary field only^21^. In particular, both amplitude and phase lag of **B**_**2**_ with respect to **B**_**1**_ will be measured, so to reconstruct the conductivity of the tissue under investigation. The relative phase, due to imaginary terms directly related to *σ* (Eq. 1), allows the creation of conductivity maps of the heart. This is, as anticipated, the most appealing advantage of OAMs-based MIT, and a fundamental prerequisite for the use of the device in the common clinical practice and in operating theatres.

The amplified signals produced by the phase-sensitive detection system are fed to a multi-channel DAQ board connected to a computer. Here, raw data are stored and processed by a dedicated software.

In view of the realisation of a practical device, it is worth noting that the OAMs array can be designed in a compact and integrated device, by exploiting also optical fibres links and integrated waveguides, thus containing the footprint. Moreover, it is important to underline that the 3D image reconstruction via the solution of the inverse problem^26^,^27^ would require a more structured array and excitation scheme than the planar geometry shown in Fig. 2.

In this case, the tissues of interest will be displayed by a computer screen as 3D conductivity maps, unlike other non-invasive mapping techniques based on electric signals^8^. In other words, a 3D representation of the heart will be provided, with local “bright” spots indicating the areas of increased conductivity. The RF ablation of such areas will produce a dramatic decrease of the local conductivity, and hence an observable change in the map. Cross-sections of the 3D map can be isolated for further diagnostic or subsequent investigation. Time-resolved maps and models can be created, in order to study the evolution of the conductivity before and after the RFCA.

### Feasibility

The feasibility of MIT measurements with an OAM was recently demonstrated^21^; promising results were obtained in terms of sensitivity, operation in unshielded environment, imaging and spatial resolution^33^. However, for the present proposal, major concerns arise about the penetration of the primary magnetic field and the tomography capability.

### Penetration capability

The skin depth, which regulates the penetration of an AC field in a medium, in the case of poor conductors at high frequency, is given by^28^:


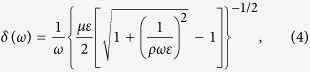


where *ω* = 2*πν*_*AC*_ is the field’s angular frequency; *μ* = *μ*_0_*μ*_*r*_ is the magnetic permeability, *ε* = *ε*_0_*ε*_*r*_ is the electric permittivity, and *ρ* = 1/*σ* is the resistivity of the medium.

**Figure 3:**
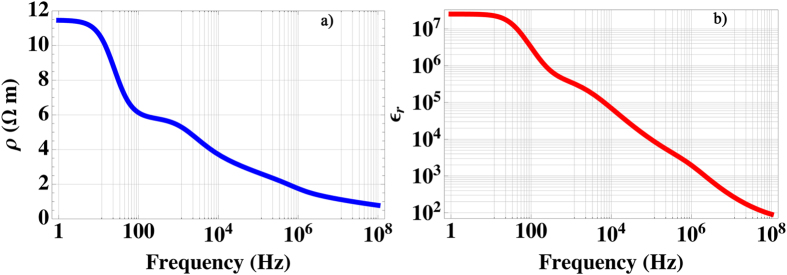
Computed resistivity (*ρ*, (a)) and relative permeability (*ε*_*r*_, (b)) of the health human cardiac tissue in the [1, 10^8^] *Hz* band.

Resistivity and relative permittivity of the human heart are here computed and shown in Fig. 3, in the band between 1 H_Z_ and 100 *MHz*, under the assumption of non-magnetic response, i.e. *μ*_*r*_ ≡ 1.

The penetration of the MIT primary field **B**_**1**_ is investigated in detail. By using the procedure described in Methods, *ρ*, *ε*_*r*_ and *δ* are computed for human tissues typically involved in the imaging of the heart: skin, fat, muscle and bone.

The resistivity of bones and fat is much larger than that of other tissues (see also Table 1), independently from the water and minerals content. In particular, there is a significant difference with respect to the heart, which implies that an almost negligible contribution, compared to that of the cardiac tissue, will be observed from those.

The relative attenuation of the MIT field **B**_**1**_ along the 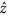 direction, ideally penetrating inside the patient’s chest is given by:


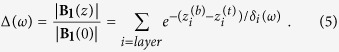


In Eq. 5, a sum is performed over the different layers of gas (i.e. air) and tissues encountered by the AC primary field traveling to the heart; each layer is located between a top position 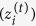 and a bottom one 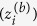, so that 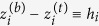 (Table 1).

**Figure 4:**
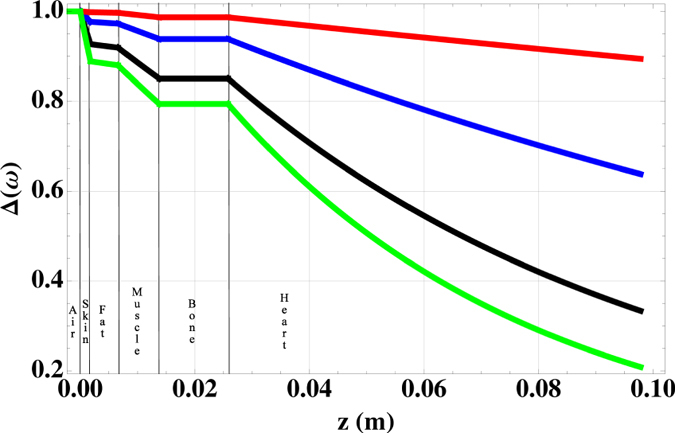
Simulation of the relative attenuation of **B**_**1**_ in the human heart’s MIT imaging. From top to bottom: red 1 *MHz*; blue 10 *MHz*; black 50 *MHz*; green 100 *MHz*.

Results shown in Fig. 4 demonstrate the feasibility of MIT measurement of the human heart.

The attenuation of the primary field becomes, as expected, larger with increasing frequency *ν*_*AC*_; however, *B*_1_ will be reduced by ~20% at the surface of the heart at 100 *MHz*, thus allowing an effective excitation of eddy currents. Furthermore, bones and fat result almost completely transparent to electromagnetic excitations in the *MHz* band. Therefore, the OAM-based MIT instrument is insensitive to screening effects, unlike purely electrical methods like the ECG, whose signal can exhibit large variations among different patients. It is worth noting also that air (at *z* < 0 in Fig. 4) is completely transparent in this band.

According to Fig. 4, the largest attenuation of the driving field is predicted within the heart itself: the instrument, consequently, will be highly sensitive to the heart’s response. In addition, this allows for a fine control of the penetration depth with the frequency *ν*_*AC*_, as described in the next section.

The secondary field *B*_2_ will experience an analogous attenuation while traveling from the heart to the sensor, given the fact that its oscillation will be at the frequency of the primary field. In addition, possible diffraction effects at the boundaries between different tissues do not represent an issue: the present approach relies on a relative measurement of the conductivity and, therefore, systematic effects due to the specificity of the patient’s anatomy will not hamper the investigation. Moreover, image reconstruction and rejection of possible artefacts can be implemented and optimised.

It is worth noting that, within the validity of Eq. 1, the conductivity term is about 5 times larger than the permittivity one at 1 *MHz* and about 3.4 times at 50 *MHz*. Therefore, although not completely negligible, spurious contributions to the secondary field will not hamper the measurement of *σ*.

### Tomography capability

**Figure 5:**
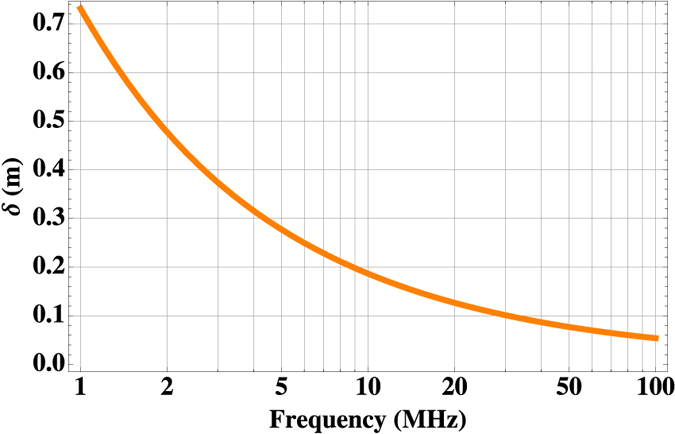
Tomography of the heart: simulation of the penetration depth in human heart, as a function of the primary field’s frequency.

By exploiting Eq. 4, the effect of frequency change on the penetration of the MIT primary field **B**_**1**_ in the heart is simulated. Results are shown in Fig. 5, in the range *ν*_*AC*_ = 1 − 100 *MHz*.

Figure 5 proves the potential for the effective tomographic operation of the proposed instrument: by selecting the frequency of **B**_**1**_, it is possible to achieve different penetrations in the patient’s heart, and, therefore, to map the cardiac tissues’ conductivity at different depths. In particular, since the tuning of *ν*_*AC*_ can be practically continuous, a fine control on the penetration depth can be achieved. This, together with the higher sensitivity to the cardiac tissue’s characteristics, makes our instrument suitable also for the localisation of abnormally conducting regions within the bulk of the heart. Incidentally, Fig. 5 further demonstrates that **B**_**2**_ can propagate throughout the bulk of the heart, which has a cross-section along 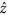 of ~7 *cm*.

These results are possible only thanks to the broad bandwidth of the OAMs’ array, by taking advantage of the OAMs’ efficient suppression of the 1/*f* noise and negligible inertia of the atomic precession. This would allow also a multi-frequency approach^11^, so to perform subtraction imaging either for the elimination of spurious contributions, such as prosthetics, reconstruction plates or other surgical devices, or for the investigation of specific phenomena and conditions.

This would not be possible, for example, with MAT-MI, where the limited bandwidth of the acoustic transducer and the sensitivity of acoustic waves to scattering by the body’s structures^16^ would limit the effective penetration and, in particular, likely hamper the investigation of the heart’s conductivity.

### Spatial and temporal resolution

Spatial resolution of the order of a few *mm* was experimentally achieved^21^. The proposed instrument hence has the potential for spatial resolution comparable or better than that of other experimental techniques such as endoscopic electrodes^7^. In addition, our device does not require the adhesion to the patient’s body or to the inner heart’s surface, with obvious advantages in terms of stability and repeatability of the measurement, and its spatial resolution does not depend on the exciting frequency, unlike the case of MAT-MI^15^.

It is worth recalling that the size of an average scarring during RF ablation is >5 *mm*: on the one hand, therefore, the spatial resolution reached by our proof-of-principle system^21^ is sufficient; on the other hand, an improvement in spatial resolution will allow a more precise ablation procedure, and ultimately a containment of collateral damage. In this view, the array arrangement and the possibility of increasing its density will have a relevant impact. Furthermore, it can be speculated that, with an optimised design of the array in view of the solution of the inverse problem, the final spatial resolution could be further improved.

Finally, given the different time scales of the processes involved, our system has the capability to reject spurious contributions due to the patient’s activity and heart’s movements. In fact, the normal heart’s beat rate can be approximated as 1 *Hz*, breath rate as 0.5 *Hz*, while the MIT OAM system will operate in the *ν*_*AC*_ = 1 *MHz* − 10^2^ *MHz* band. Hence, to a first approximation, the heart’s natural motion will not be a limiting factor for the spatial resolution and suitable averaging sequences can be implemented. In particular, the minimum time scale of the OAMs is of the order of 2*π*/*ω*_*L*_; in the case of the MIT operation, this lower limit can be estimated as 1/*ν*_*AC*_. Both are much shorter than the average duration of the involved biological processes.

## Discussion

The proposed application is technically feasible only thanks to the use of the optical atomic magnetometers’ array; however, the OAMs’ characteristics will also provide several advantages also in the daily clinical practice. The direct mapping of the heart’s conductivity, indeed, allows to address simultaneously the anatomy, the physiology and the pathology of the heart, thus filling up the lack of dedicated diagnostic tools.

The approach is completely non-invasive and harmless for the patient and the operator: AC magnetic fields will be roughly 10^5^ times smaller than the typical Magnetic Resonance Imaging (MRI) field. Moreover, unlike MRI, our system is inherently safe also for other devices, such as diagnostic and medical (e.g. pace-makers) equipment.

Unlike ECG, the OAM MIT will not be affected by the body’s screening effects or contingent anomalies in the environment; it will not require contact with the patient, which can be important in the case of infective diseases, extended wounds, or burns on the chest. Furthermore, unlike SQUIDs, the system can be operated in unshielded environments and at room temperature; it has indeed the potential for actual bedside operation.

The diagnosis of fibrillation and its monitoring during surgery currently require an ongoing crisis; hence, for example, AF is induced during the RFCA procedure^6^,^7^,^8^. Our instrument, instead, is capable of detecting the sustaining structures even in absence of a fibrillation event. Novel procedures can thus be envisioned and large scale screening campaigns can be designed, as well as event-triggered monitors, for example with standard ECG, in order to get more insight on the fundamental mechanisms leading to AF or VF.

At the same time, the OAM MIT provides a new tool for: i. assessment of patients suitable for surgery, ii. surgery planning and design, and iii. surgery navigation and control, without the need of X-ray based imaging techniques, such as fluoroscopy, and with minimum occupation of the surgical field.

Finally, the proposed instrument is suitable also for the investigation of other body’s tissues and the diagnosis of specific conditions, where conductivity is, or is expected to be, an important parameter. Among the possible examples: peripheral nerves in neurodegenerative conditions, optic nerve in optic neuropathy and neuritis, skin healing after grafts, burns, or wounds, and detection of anomalous structures, such as early stage melanomas. Moreover, indications of a correlation between specific tumours and change of the electric properties of the malignant tissue were found in the context of RF-induced hyperthermia: with a cancer, the conductivity of liver and lung tissues increases by more than 30%, and by more than 500% in the case of mammary tissue^29^. If such data are confirmed, non-invasive maps of tissues’ conductivity will likely provide an early diagnosis and localisation of tumours, with a potential dramatic impact on the treatments’ effectiveness.

In conclusion, MIT is here identified as the ideal technique for shading new light on cardiac fibrillation. Therefore, an instrument based on an array of optical atomic magnetometers for magnetic induction tomography of heart is proposed. MIT allows the non-invasive and harmless mapping of the heart’s conductivity. OAMs overcome the limitation of the current MIT instrumentation, mainly in terms of sensitivity, footprint, scalability and flexibility. In addition, the capability of OAMs of operation at room temperature and without dedicated shielding will ease the transfer of the technology from the laboratory to the clinical environment.

Our results indicate that the system will have the capability to operate in the required regime, with sufficient spatial resolution and in a real tomography mode, by simply adjusting the driving frequency. The merge of MIT and OAMs will thus provide a novel diagnostic tool, with no equal currently available. The device will improve the patients’ management in the case of heart conditions, by offering new research and clinical solutions, and the possibility of an effective patient-tailored diagnostic technique. This will increase the success rates of the treatment of fibrillations^6^ and clarify their underlying mechanisms.

Finally, the device is a promising asset also for investigations on the conductivity anomalies in other conditions of the nerves, skin and other organs and tissues, such as liver and breast. A relevant impact on the early diagnosis and localisation of peripheral neuropathies, structural anomalies, degenerative conditions and tumours can be therefore envisioned.

## Methods

### Experimental design: RF OAM for MIT

The atomic vapour is contained in cubic glass cells, so to allow optical access from four sides; a naturally occurring isotopic mixture of Rb can be used, with a few *Torr* of a noble buffer gas, in order to increase the interaction time and reduce the depolarising atom/wall collisions.

A distributed Bragg reflector laser, chosen in order to achieve the maximum stability and portability, is tuned to the *F* = 2 → *F*′ = 3 hyperfine transition of ^87^Rb D_2_ line, at 780 nm. The laser beam is split in a pair of pump and probe beams for each cell of the array. The pump is circularly polarised (*σ*^+^). A pair of Helmholtz coils with the axis parallel to **k**_**pump**_, the wavevector of the pump beam, allows optical pumping to the |*F* = 2, *m*_*F*_ = +2〉 Zeeman sub-level of the ^87^Rb ground state. Consequently, long-living atomic orientation of the ground state is created.

The probe beam is linearly polarised (*π*) perpendicularly to **k**_**pump**_ and **k**_**probe**_, the wavevectors of pump and probe laser beams, respectively. The beams are crossed at the centre of each cell. The actual size of the sensor is given by the overlapping of the two beams and, thus, can be easily adapted to the experimental requirements,also in view of the miniaturisation of the device. By assuming a beam waist of 4 *mm* in the centre of each vapour cell, about 210 *μW* of circularly polarised light are sufficient to saturate the transition 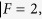 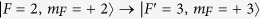
30. The probe beam, consistently, should have a power of the order of a few tens of *μW*: the minimum total power requirement for each cell in the OAMs’ array is therefore ~250 *μW*. This implies that a large array can be easily driven by a single laser source.

A radio-frequency magnetic field, perpendicular to the plane where **k**_**pump**_ and **k**_**probe**_ lie, drives coherently the ground-state population’s polarisation. In view of applications, with a suitable arrangement, the RF coil can be integrated with the MIT primary coil^33^.

### Dielectric properties of thoracic tissues

Dielectric properties of biological tissues are the result of the forced motion of electrolytes in the intra- and intercellular volumes. In particular, in the *MHz* band, the conductivity is mainly determined by mobility and molar concentration of ions. Experimental data reported in literature about biological tissues *in vivo* are often incomplete, or exhibit large fluctuations. Therefore, the frequency dependence of *ρ* and *ε*_*r*_ is here computed with the Cole-Cole equation^31^:


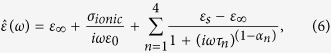


where *ε*_∞_ is the electric permittivity at high frequencies 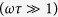, while *ε*_*s*_ is the quasi-static permittivity 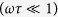; *σ*_*ionic*_ is the static ionic conductivity. *τ*_*n*_ is *n* − *th* relaxation time and {*α*_*n*_} is a set of phenomenological parameters describing the broadening of the *n* − *th* contribution. In fact, the tissues’ response to oscillating electric and magnetic fields is empirically characterised by four regions with different polarisation characteristics, in particular with different relaxation times, *τ*_*n*_.

From Eq. 6, *ρ*(*ω*) and *ε*_*r*_(*ω*) can be directly computed:


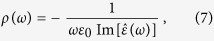



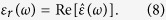


In order to obtain a more consistent estimation of *ρ*(*ω*), the frequency dependence, given by Eq. 7, is re-scaled with the average values reported in literature^32^. In the case of relative permittivity *ε*_*r*_, to the best of our knowledge, such comprehensive investigations cannot be found. Therefore, data as obtained by Eq. 8 are taken into consideration.

Results concerning tissues located in the human chest are summarised in Table 1, at 50 *MHz*.

## Additional Information

**How to cite this article**: Marmugi, L. and Renzoni, F. Optical Magnetic Induction Tomography of the Heart. *Sci. Rep.* **6**, 23962; doi: 10.1038/srep23962 (2016).

## Figures and Tables

**Table 1:** Computed *ρ*, *ε*_*r*_, *δ* at 50 *MHz* and expected thickness (*h*) of the human tissues surrounding the heart.

Tissue	*ρ*(Ω*m*)	*ε*_*r*_	*δ*(*m*)	*h*(*mm*)
Skin (dry)	0.11	107.1	0.024	1.8
Fat	26.2	14.5	0.58	4.8
Muscle	1.3	71.3	0.28	7.2
Bone	5.3 × 10^5^	17.7	≫10	12 (sternum)

## References

[b1] Longo D. *et al.* Harrison’s Principles of Internal Medicine 18th edn, Vol. 2, Ch. 233, 3851–3856 (McGraw Hill Professional, 2012).

[b2] Vaquero M., Calvo D. & Jalife J. Cardiac fibrillation: from ion channels to rotors in the human heart. Heart Rhythm. 5, 872–879, 10.1016/j.hrthm.2008.02.034 (2008). 18468960 PMC2486257

[b3] Christ T. *et al.* Arrhythmias, elicited by catecholamines and serotonin, vanish in human chronic atrial fibrillation. Proceedings of the National Academy of Sciences 111, 11193–11198, 10.1073/pnas.1324132111 (2014). PMC4121801 25024212

[b4] Narayan S. M., Patel J., Mulpuru S. & Krummen D. E. Focal impulse and rotor modulation ablation of sustaining rotors abruptly terminates persistent atrial fibrillation to sinus rhythm with elimination on follow-up: A video case study. Heart Rhythm. 9, 1436–1439, 10.1016/j.hrthm.2012.03.055 (2012). 22465458 PMC3432749

[b5] Ganesan A. N. *et al.* Long-term outcomes of catheter ablation of atrial fibrillation: A systematic review and meta-analysis. Journal of the American Heart Association 2, 10.1161/JAHA.112.004549 (2013). PMC3647286 23537812

[b6] Sanders P. *et al.* Spectral analysis identifies sites of high-frequency activity maintaining atrial fibrillation in humans. Circulation 112, 789–797, 10.1161/CIRCULATIONAHA.104.517011 (2005). 16061740

[b7] Narayan S. M., Krummen D. E. & Rappel W.-J. Clinical mapping approach to diagnose electrical rotors and focal impulse sources for human atrial fibrillation. Journal of cardiovascular electrophysiology 23, 447–454, 10.1111/j.1540-8167.2012.02332.x (2012) 22537106 PMC3418865

[b8] Haissaguerre M. *et al.* Noninvasive panoramic mapping of human atrial fibrillation mechanisms: A feasibility report. Journal of Cardiovascular Electrophysiology 24, 711–717, 10.1111/jce.12075 (2013). 23373588

[b9] Xie W. *et al.* Mitochondrial oxidative stress promotes atrial fibrillation. Scientific Reports 5, 11427 EP-, 10.1038/srep11427 (2015). 26169582 PMC4501003

[b10] Griffiths H. Magnetic induction tomography. Measurement Science and Technology 12, 1126, 10.1088/0957-0233/12/8/319 (2001).

[b11] Darrer B. J., Watson J. C., Bartlett P. & Renzoni F. Magnetic imaging: a new tool for UK national nuclear security. Sci. Rep. 5, 2271 EP-, 10.1038/srep07944 (2015). PMC4302293 25608957

[b12] Ma X., Peyton A. J., Higson S. R. & Drake P. Development of multiple frequency electromagnetic induction systems for steel flow visualization. Measurement Science and Technology 19, 094008, 10.1088/0957-0233/19/9/094008 (2008).

[b13] Zolgharni M., Griffiths H. & Ledger P. D. Frequency-difference MIT imaging of cerebral haemorrhage with a hemispherical coil array: numerical modelling. Physiological Measurement 31, S111, 10.1088/0967-3334/31/8/S09 (2010). 20647622

[b14] Griffiths H., Stewart W. R. & Gough W. Magnetic induction tomography: A measuring system for biological tissues. Annals of the New York Academy of Sciences 873, 335–345, 10.1111/j.1749-6632.1999.tb09481.x (1999). 10372181

[b15] Sun X., Fang D., Zhang D. & Ma Q. Acoustic dipole radiation based electrical impedance contrast imaging approach of magnetoacoustic tomography with magnetic induction. Medical Physics 40, 052902, 10.1118/1.4800639 (2013). 23635295

[b16] Mariappan L., Hu G. & He B. Magnetoacoustic tomography with magnetic induction for high-resolution bioimepedance imaging through vector source reconstruction under the static field of mri magnet. Medical Physics 41, 022902, 10.1118/1.4862836 (2014). 24506649 PMC3987704

[b17] Budker D. & Romalis M. Optical magnetometry. Nat Phys 3, 227–234, 10.1038/nphys566 (2007).

[b18] Kominis I. K., Kornack T. W., Allred J. C. & Romalis M. V. A subfemtotesla multichannel atomic magnetometer. Nature 422, 596–599, 10.1038/nature01484 (2003). 12686995

[b19] Schwindt P. D. D. *et al.* Chip-scale atomic magnetometer. Applied Physics Letters 85, 6409–6411, 10.1063/1.1839274 (2004).

[b20] Savukov I., Seltzer S. & Romalis M. Detection of NMR signals with a radio-frequency atomic magnetometer. Journal of Magnetic Resonance 185, 214–220, 10.1016/j.jmr.2006.12.012 (2007). 17208476

[b21] Wickenbrock A., Jurgilas S., Dow A., Marmugi L. & Renzoni F. Magnetic induction tomography using an all-optical ^87^Rb atomic magnetometer. Opt. Lett. 39, 6367–6370, 10.1364/OL.39.006367 (2014). 25490470

[b22] Smullin S. J., Savukov I. M., Vasilakis G., Ghosh R. K. & Romalis M. V. Low-noise high-density alkali-metal scalar magnetometer. Phys. Rev. A 80, 033420, 10.1103/PhysRevA.80.033420 (2009).

[b23] Belfi J., Bevilacqua G., Biancalana V., Dancheva Y. & Moi L. All optical sensor for automated magnetometry based on coherent population trapping. J. Opt. Soc. Am. B 24, 1482–1489, 10.1364/JOSAB.24.001482 (2007).

[b24] Ledbetter M. P., Savukov I. M., Acosta V. M., Budker D. & Romalis M. V. Spin-exchange-relaxation-free magnetometry with Cs vapor. Phys. Rev. A 77, 033408, 10.1103/PhysRevA.77.033408 (2008).

[b25] Pustelny S. *et al.* Magnetometry based on nonlinear magneto-optical rotation with amplitude-modulated light. Journal of Applied Physics 103, 063108, 10.1063/1.2844494 (2008).

[b26] Merwa R., Hollaus K., Brunner P. & Scharfetter H. Solution of the inverse problem of magnetic induction tomography (MIT). Physiological Measurement 26, S241, 10.1088/0967-3334/26/2/023 (2005). 15798237

[b27] Wei H.-Y., Ma L. & Soleimani M. Volumetric magnetic induction tomography. Measurement Science and Technology 23, 055401, 10.1088/0957-0233/23/5/055401 (2012).

[b28] Griffiths D. J. Introduction to electrodynamics 3rd edn, Ch. 9, 392–405 (Prentice Hall Upper Saddle River, NJ, 1999).

[b29] Joines W. T., Zhang Y., Li C. & Jirtle R. L. The measured electrical properties of normal and malignant human tissues from 50 to 900 MHz. Medical Physics 21, 547–550, 10.1118/1.597312 (1994). 8058021

[b30] Steck D. A. Rubidium 87 D line data (2010). URL http://steck.us/alkalidata.

[b31] Gabriel S., Lau R. W. & Gabriel C. The dielectric properties of biological tissues: III. parametric models for the dielectric spectrum of tissues. Physics in Medicine and Biology 41, 2271, 10.1088/0031-9155/41/11/003 (1996). 8938026

[b32] Faes T. J. C., van der Meij H. A., de Munck J. C. & Heethaar R. M. The electric resistivity of human tissues (100 Hz–10 MHz): a meta-analysis of review studies. Physiological Measurement 20, R1, 10.1088/0967-3334/20/4/201 (1999). 10593226

[b33] Deans C., Marmugi L., Hussain S. & Renzoni F. Electromagnetic induction imaging with a radio-frequency atomic magnetometer. Applied Physics Letters 108, 103503, 10.1063/1.4943659 (2006).

